# Case Report: Deep brain stimulation in SYNJ1-related early-onset parkinsonism

**DOI:** 10.3389/fmed.2026.1895449

**Published:** 2026-07-13

**Authors:** Jiali Liu, Youcheng Zhang, Shouxuan Chen, Bin Xu, Zhengzheng Huang, Liping Zhou, Xin Zheng

**Affiliations:** 1 Department of Neurosurgery, Shenzhen Key Laboratory of Neurosurgery, The First Affiliated Hospital of Shenzhen University, Shenzhen Second People's Hospital, Shenzhen, China; 2 Faculty of Synthetic Biology, Shenzhen Institute of Advanced Technology, Chinese Academy of Sciences, Shenzhen, China; 3 Department of Neurology, South China Hospital of Shenzhen University, Shenzhen, China; 4 Department of Neurology, The First Affiliated Hospital of Shenzhen University, Shenzhen Second People's Hospital, Shenzhen, China; 5 Research Centre for Chinese Medicine Innovation (RCMI), The Hong Kong Polytechnic University, Kowloon, Hong Kong SAR, China

**Keywords:** atypical Parkinson’s disease, monogenic Parkinsonisms, early-onset parkinsonism, *SYNJ1* gene, deep brain stimulation

## Abstract

**Background:**

Deep brain stimulation (DBS) is established for levodopa-responsive Parkinson’s disease (PD), but the outcomes of DBS in rare genetic early-onset parkinsonism remain incompletely defined. While its efficacy is documented in monogenic forms of parkinsonism (e.g., *LRRK2*, *Parkin*, *PINK1,* and *SNCA*), clinical data regarding the safety and efficacy of DBS in carriers of the *SYNJ1* mutation remain scarce.

**Case presentation:**

We report two patients with *SYNJ1*-related early-onset parkinsonism treated with bilateral DBS and place their outcomes in a single-center gene-listed EOPD DBS cohort. Case 1 carried a novel homozygous SYNJ1 c.1969A > G (p. Thr657Ala) missense variant and 46, XY disorder of sex development; GPi-DBS enabled the levodopa equivalent daily dose (LEDD) reduction from 775 to 300 mg/day, with improved dyskinesia and sleep, although motor fluctuations and neuropsychiatric symptoms persisted. Case 2 carried a SYNJ1 c.1627 + 13 T > A intronic variant; subthalamic nucleus deep brain stimulation (STN-DBS) reduced the LEDD from 955 to 605 mg/day and Unified Parkinson’s Disease Rating Scale (UPDRS-III) score from 51 to 32 at 1.5-year follow-up. In the contextual cohort, the median LEDD reduction after DBS was 35.2%. These observations support DBS as a feasible symptomatic strategy in selected genetic early-onset Parkinson’s disease (EOPD) while emphasizing genotype-specific uncertainty.

**Conclusion:**

These cases illustrate heterogeneous clinical responses to DBS in patients with early-onset parkinsonism carrying SYNJ1 variants. DBS may provide symptomatic benefit in carefully selected patients, but the small sample size and variant-level uncertainty preclude conclusions regarding genotype-specific efficacy.

## Introduction

Early-onset Parkinson’s disease (EOPD) is enriched for genetic susceptibility and often exposes patients to many years of dopaminergic therapy, motor fluctuations, dyskinesia, and disability. Genetic diagnosis is increasingly relevant in EOPD because it informs counseling, phenotypic interpretation, family screening, and trial readiness; however, genotype-specific evidence for device-aided treatment remains limited (1, 2). Over the past 25 years, genetic research has identified 23 loci and 13 genes firmly associated with inherited parkinsonism (3). Among these, 10 genes, namely *PRKN*, *PINK1*, *DJ-1*, *ATP13A2*, *PLA2G6*, *FBXO7*, *DNAJC6*, *SYNJ1*, *VPS13C*, and *PTRHD1*, are typically linked to levodopa-responsive early-onset (EO) PD with slow progression. However, specific rare mutations in genes such as *SYNJ1* and *DNAJC6* can lead to more severe phenotypes characterized by gaze palsy, dementia, and epileptic encephalopathy (4).

SYNJ1 encodes synaptojanin 1, a phosphoinositide phosphatase involved in synaptic vesicle endocytosis (5). Biallelic SYNJ1 variants cause PARK20, a rare autosomal-recessive early-onset parkinsonism with broad clinical variability ranging from levodopa-responsive parkinsonism to complex atypical syndromes with seizures, cognitive impairment, gaze palsy, or other neurological features (4, 6, 7). Published reports have mainly described natural history and genotype–phenotype relationships rather than surgical outcomes.

Deep brain stimulation (DBS) is a standard intervention for advanced PD, typically targeting the subthalamic nucleus (STN) or the globus pallidus internus (GPi), to improve motor function and reduce dopaminergic requirements (8). While its efficacy in monogenic forms such as *GBA*, *LRRK2,* and *Parkin* is well-documented (9–11), there is a complete lack of data on DBS outcomes in *SYNJ1* carriers. Furthermore, most reported variants are located in exonic regions or canonical splice sites, while deep intronic variants are rarely reported. In this report, we present the clinical course of two PARK20 cases carrying distinct variants, detailing the 1.5-year post-operative outcomes of bilateral DBS and highlighting their impacts on genotype–phenotype correlations. In addition, we contextualize these outcomes using a single-center gene-listed EOPD DBS cohort.

## Results

### Case 1: novel homozygous SYNJ1 missense variant treated with GPi DBS

A 45-year-old woman, born to consanguineous parents and possessing a 46, XY karyotype, presented with rapidly progressing early-onset Parkinson’s disease (EOPD). Her symptoms began at age 41 with right-hand rigidity and hyposmia, followed by gait impairment, reduced arm swing, shortened stride length, sleep disturbance, end-of-dose wearing off, on–off fluctuations, nocturnal dyskinesia, and off dystonia. Preoperative UPDRS-III score improved from 47 (“off”) to 15 (“on”), indicating a 68.1% response to levodopa. Cognitive function remained intact, with a Mini-Mental State Examination (MMSE) score of 30/30 and an education-adjusted Montreal Cognitive Assessment (MoCA) score of 29/30 (Table 1).

**Table 1:** Pre-operative and post-operative drug efficacy and PD-related scale results.

Clinical Scale/Assessment item	Pre-operative	Post-operative (1.5 years after DBS)
Drug prescription	Levodopa/benserazide 125 mg qid + qn Selegiline 5 mg bid Pramipexole 0.25 mg bid+qn	Levodopa/benserazide 125 mg qid Entacapone 0.1 g tid Piribedil 50 mg qd + qn Olanzapine 25 mg qn
Levodopa equivalent dose	775 mg	300 mg
Hoehn and Yahr stages	2.5	2.5
MDS-UPDRS scores		
Part I	2 + 7	8 + 5
Part II	14	15
Part III	47 (med-off)	57 (stim-on med-off)
	15 (med-on)	63 (stim-off med-off)
		41 (stim-off med-on)
		58 (stim-on med-failed)
Part IV	10	7
PDQ-39	45/156	46/156
Non-motor scales		
MMSE	30/30	30/30
MoCA	28 + 1/30 (with 8-year education)	26 + 1/30
HAMA	12/56 (might have anxiety)	26/56 (obvious anxiety)
HAMD-24	14/96 (might have depression)	9/96 (might have depression)
BDI-II	10/63 (minimal depression)	12/63 (minimal depression)
PSQI	16 (sleep disturbance)	10 (sleep disturbance)
PDSS	106/150 (possible sleep problem)	127/150 (uncertain)
ESS	2/24 (normal)	3/24 (normal)
NMSS	Sleep: RLS, easy awakening Mood: apathy, depression, anxiety	Mood: apathy, depression

MDS-UPDRS, The Movement Disorder Society-Sponsored Revision of the Unified Parkinson’s Disease Rating Scale; med-off, medication “off” status; med-on, medication “of” status; stim-off, stimulation “off” status; stim-on, stimulation “on” status; PDQ-39, The Parkinson’s Disease Questionnaire; MMSE, Mini-Mental State Examination; MoCA, Montreal Cognitive Assessment; HAMA, Hamilton Anxiety Rating Scale; HAMD, Hamilton Rating Scale for Depression; BDI, Beck Depression Inventory; PSQI, The Pittsburgh Sleep Quality Index; PDSS, Parkinson’s disease sleep scale; ESS, The Epworth sleepiness scale; NMSS, The Non-Motor Symptoms Scale; RLS, Restless legs syndrome.

**Figure 1:**
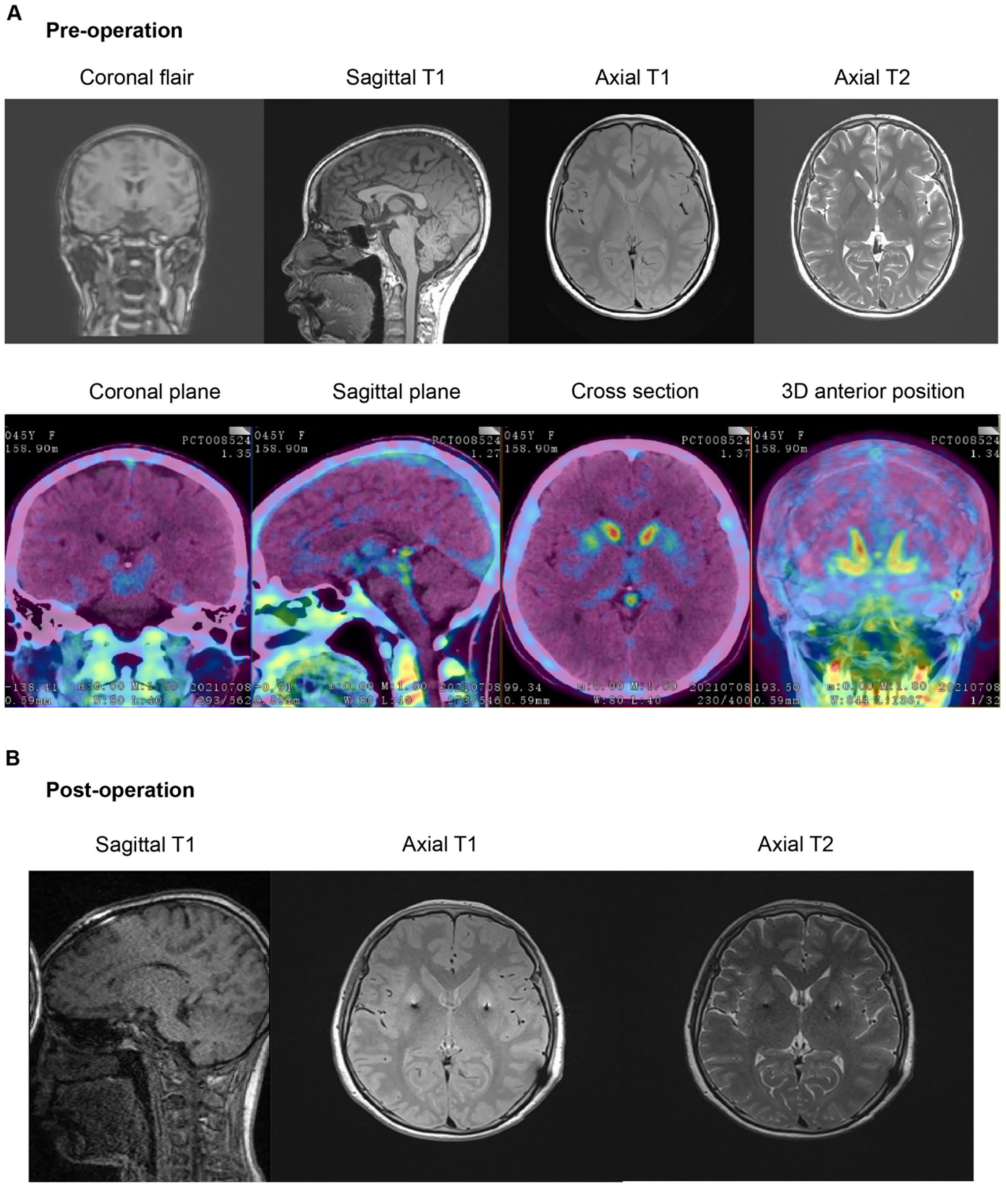
Preoperative and postoperative neuroimaging results in Case 1. **(A)** Preoperative brain magnetic resonance and PET/CT neuroimaging results. PET/CT examination showed that 18F-DOPA uptake was significantly reduced in the bilateral substantia nigra, caudate nucleus, and lentiform nucleus, especially in the bilateral lentiform nucleus. **(B)** MRI on post-operation. Axial T1 and T2 Flair-weighted MRI sequences showing postoperative images of the brain parenchyma and basal ganglia.

**Figure 2:**
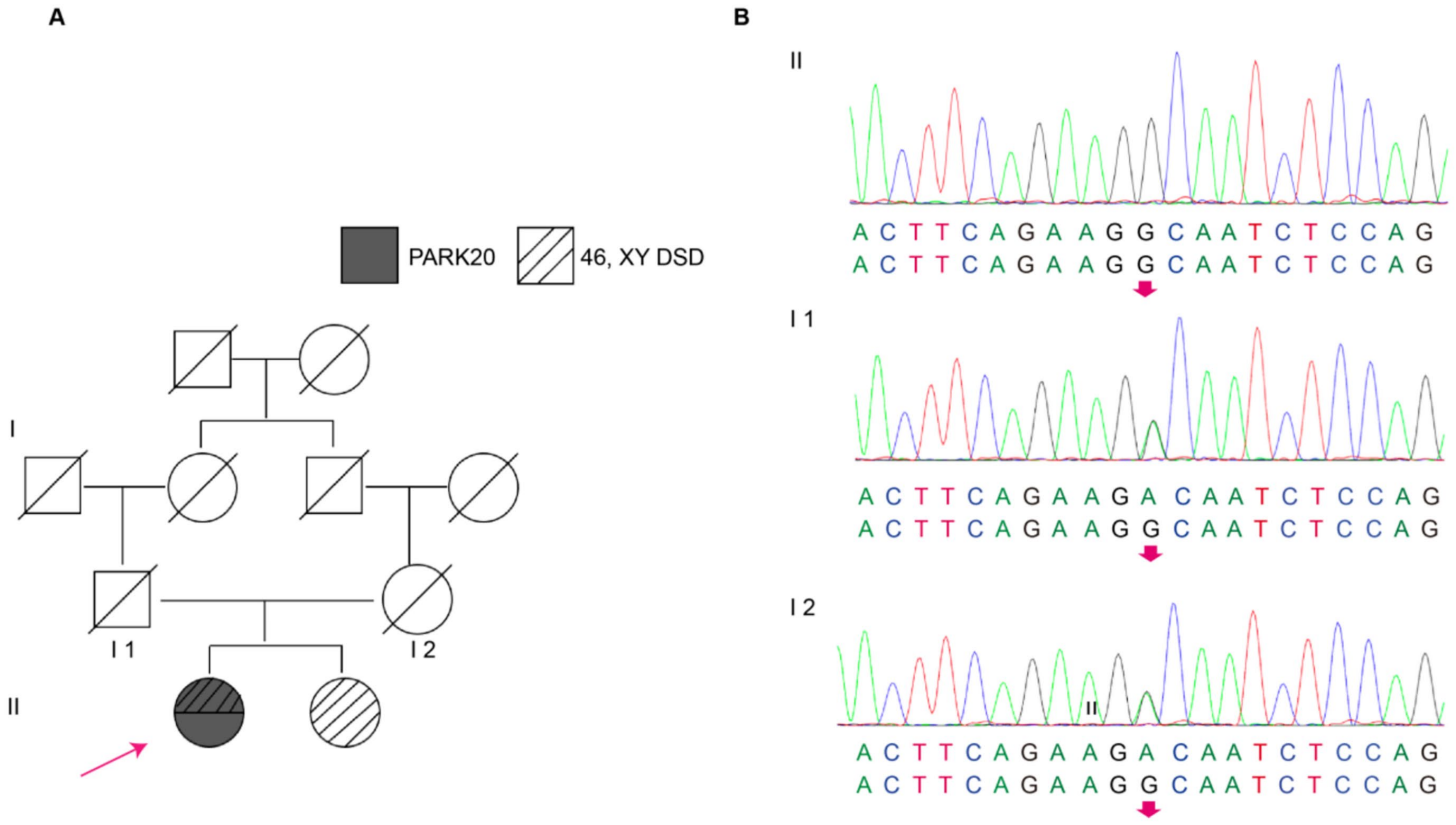
Pedigree of the patient’s family. **(A)** The patient’s family lineage gene mutation. Electropherogram of the Sanger sequencing of the mutation. **(B)** Pedigree of the patient’s family. Cycle indicates woman. Square indicates man. The dark filled symbols represent the PARK20 individuals, the light filled symbols represent the 46, XY DSD individuals, and the blank symbols represent the unaffected individuals. Double lines indicate consanguineous parent.

**Figure 3:**
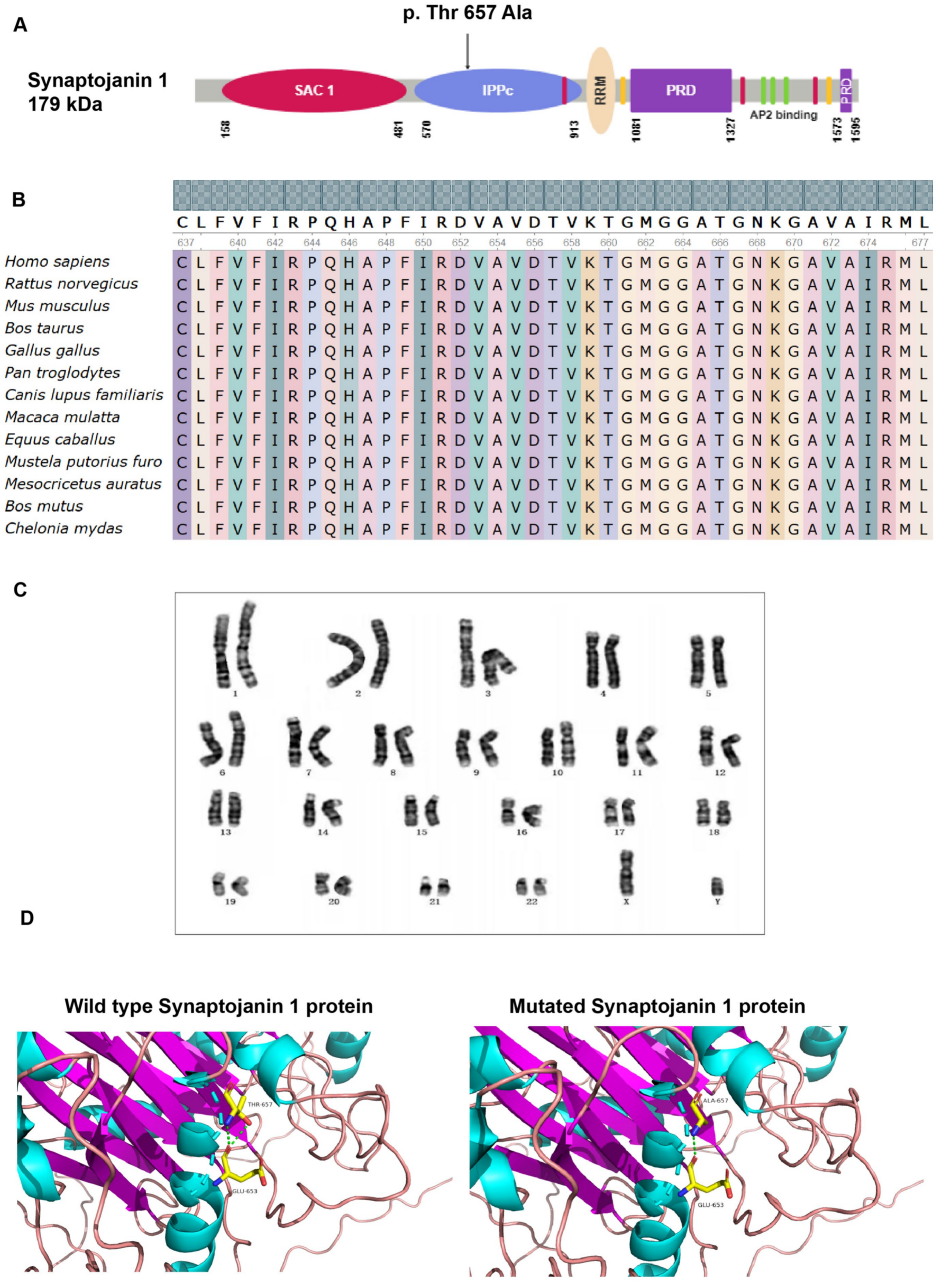
SYNJ1 c.1969A>G (p. Thr657Ala) substitution in our patient. **(A)** Schematic representation of the synaptojanin 1 protein domains and the position of the substitution. SAC1: suppressor-of-actin1-like phosphatase domain. **(B)** The alignment of the Synaptojanin 1 protein across various species. **(C)** Routine peripheral blood karyotype indicated that the patient had a 46, XY chromosomal configuration. **(D)** The 3D structure of the Synaptojanin 1 protein.

We conducted pre- and post-operative brain magnetic resonance and ^18^F-DOPA PET-CT revealed significantly reduced dopamine transporter binding in the nigrostriatal system, particularly within the striatum (Figure 1A). With no “red flag” or exclusion criteria present, she was diagnosed with clinically established Parkinson’s disease. Comprehensive Sanger sequencing identified a novel homozygous missense mutation in the *SYNJ1* gene (NM_003895.3: c.1969A > G, p.Thr657Ala) (Figure 2A). This missense mutation was also present in the unaffected parents (Figure 2B). The alignment of the Synaptojanin 1 protein across various species highlights the conservation of residue 1321, with the mutated amino acid indicated within a frame (Figure 3). Routine G-band karyotyping of peripheral blood, performed at 400-band resolution based on ISCN 2020, confirmed a male karyotype, 46, XY (Figure 3C). Both the patient and her sister exhibited a 46, XY karyotype with a female phenotype. The patient developed an infantile uterus and experienced primary amenorrhea. We used swiss-model to construct the protein model, and used pymol software for visualization analysis. The double bond in the wild-type hydrogen bond within the protein structure is converted into a single bond in the p. Thr657Ala mutated protein (Figure 3D).

Bilateral DBS electrodes (PINS L302, Beijing, China) were implanted into the GPi (Figure 1B) using Robotic Stereotactic Assistance (ROSA, MedTech Surgical, Inc., Warsaw, Indiana, USA) under general anesthesia. A pulse generator (G102RZ, PINS, Beijing, China) was subcutaneously implanted in the left sub-clavicular region. DBS programming was commenced 1-month post-operatively in the medication-off state using a monopolar review strategy. Initial parameters were set at a pulse width of 90 μs and a frequency of 150 Hz, with the voltage increasing incrementally from 0 V in 1 V step to determine the therapeutic window. Optimal contacts were selected to maximize motor benefits while minimizing side effects. The final DBS settings were as follows: left-GPi (C + 6-) 60 μs, 150 Hz, 1.5 V and right-GPi (C + 2-) 90 μs, 150 Hz, 1.5 V.

At the 1.5-year assessment, the MDS-UPDRS III scores were 63 in the stimulation-OFF/medication-OFF state, 57 in the stimulation-ON/medication-OFF state, 41 in the stimulation-OFF/medication-ON state, and 58 in the stimulation-ON/ medication-OFF state. These scores did not show consistent objective motor improvement compared to the preoperative assessments, indicating a limited and complex motor response. Nevertheless, the LEDD decreased from 775 to 300 mg/day, the UPDRS-IV score decreased from 10 to 7, dyskinesia-related items improved, and sleep measures improved (Table 1).

### Case 2: SYNJ1 intronic variant treated with STN DBS

A man with motor onset at age 44 in 2015 underwent bilateral subthalamic nucleus deep brain stimulation (STN-DBS) on 30 December 2021, after an approximately 6-year disease duration. His phenotype was predominantly akinetic-rigid. His preoperative LEDD was 955 mg/day. Baseline assessments included an MMSE score of 30/30 (6.5 years of education), a MoCA score of 29/30, 17 on the Hamilton Anxiety Scale (HAMA), 22 on the Hamilton Depression Scale (HAMD), and 65 on the Parkinson’s Disease Questionnaire (PDQ-39). Genetic testing identified a deep intronic variant c.1627 + 13 T > A in intron 12 of the *SYNJ1* gene. STN was selected because of the akinetic-rigid symptom profile, medication burden, age of 50 years at surgery, and absence of dyskinesia or cognitive decline. At 1.5 years after STN-DBS, the LEDD was reduced to 605 mg/day, and the final stimulation settings were as follows: left STN C + 6-, 3.15 V, 70 μs, 105 Hz and right STN C + 2-, 3.3 V, 70 μs, 105 Hz. The UPDRS-III score improved by 45.1% from 51 to 28 [medication state/stimulation state], indicating clinically meaningful DBS responsiveness.

### Contextual single-center gene listed EOPD DBS cohort

To place the two SYNJ1 cases in a broader clinical context, we summarized a single-center gene-listed EOPD DBS dataset. The contextual dataset contains 15 patients with motor onset at or before 50 years of age and lists gene findings (Table 2). The median age at onset was 35.0 years, the median age at DBS was 42.0 years, and the median disease duration before DBS was 6.0 years. Twelve patients received STN-only DBS, and three received GPi-only DBS.

**Table 2:** Contextual single-center gene-listed EOPD DBS cohort.

Measure	Value
Cohort size	15 gene-listed EOPD patients treated with DBS
Age at onset	Median 35.0 years (IQR 32.5–39.0)
Age at DBS	Median 42.0 years (IQR 39.5–46.0)
Disease duration before DBS	Median 6.0 years (IQR 4.5–8.0)
Sex	12 male and 3 female
DBS target	12 STN-only and 3 GPi-only
Preoperative LEDD	Median 800.0 mg/day (IQR 525.0–977.5)
Postoperative LEDD change	Median 800.0 to 530.0 mg/day; median reduction 35.2%; p = 0.014
H&Y ON stage	Median 3.0 before DBS to 2.0 after DBS; p = 0.007
Preoperative MDS-UPDRS III ON/OFF response	Median OFF 51.0 to ON 18.0; median improvement 63.3%; p = 0.002
Core PD/parkinsonism genes listed	GBA, LRRK2, PRKN, PLA2G6, ATP13A2, VPS35
Other/candidate genes listed	ABCA7, AMPD1, DYNC1H1, GIGYF2, NOS3, PNKD, SETX, TRPM2, TRPM7

**Figure 4:**
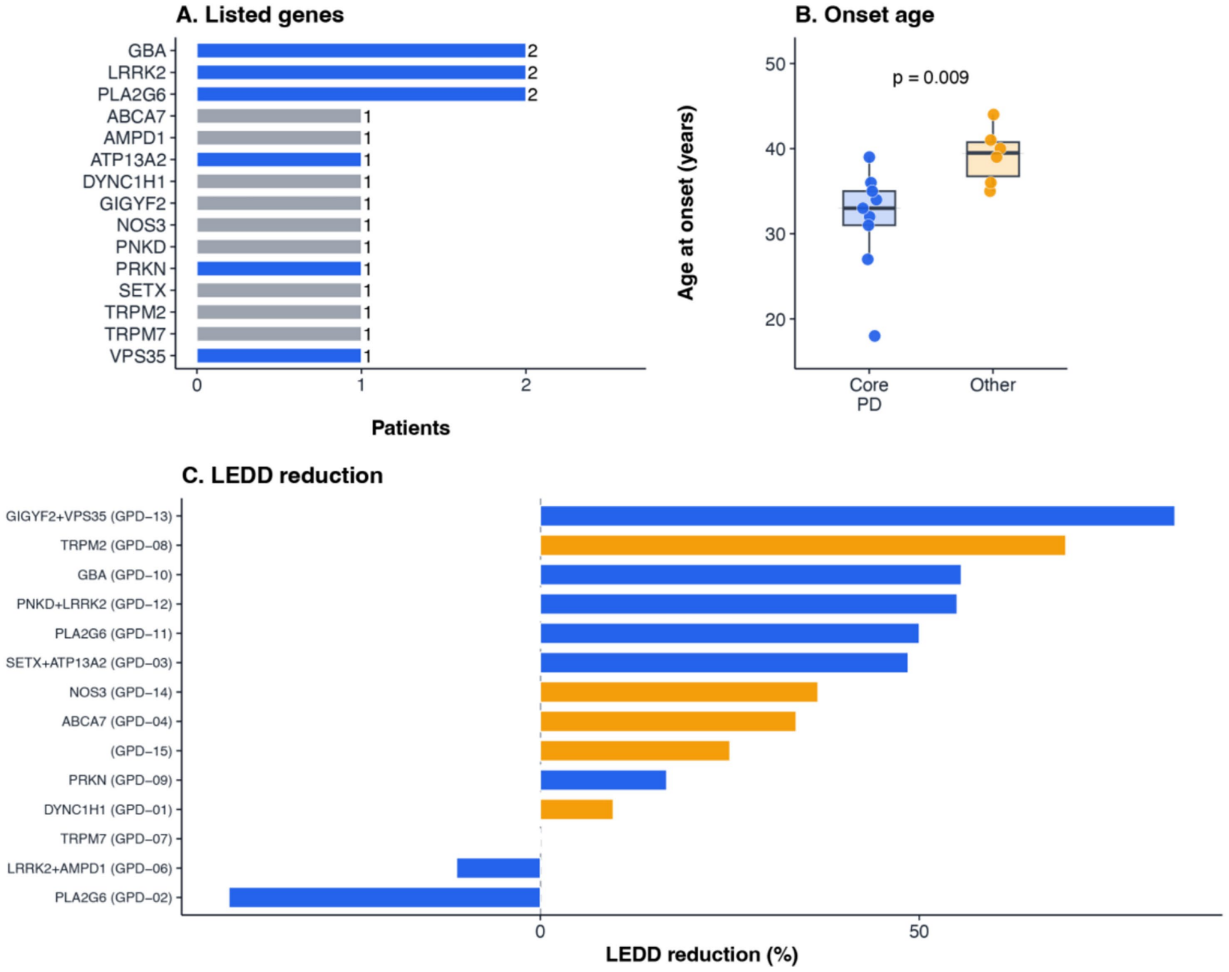
Gene spectrum and exploratory cohort findings. **(A)** Listed gene counts; **(B)** onset age by core versus other/candidate gene category. Core PD/parkinsonism genes listed in the source dataset included GBA, LRRK2, PRKN, PLA2G6, ATP13A2, and VPS35, other listed genes included ABCA7, AMPD1, DYNC1H1, GIGYF2, NOS3, PNKD, SETX, TRPM2, and TRPM7. **(C)** Patient LEDD reduction level.

Across the cohort, 18 gene entries were recorded. Core PD/parkinsonism genes listed in the source dataset included *GBA, LRRK2, PRKN, PLA2G6, ATP13A2,* and *VPS35*; other listed genes included *ABCA7, AMPD1, DYNC1H1, GIGYF2, NOS3, PNKD, SETX, TRPM2,* and *TRPM7* (Figure 4A, Supplementary Table S1). Exploratory subgroup analysis suggested that patients with a listed core PD/parkinsonism gene had an earlier onset than those with only other or candidate genes (median 33.0 versus 39.5 years; *p* = 0.009, Figure 4B). Patient-level reductions in the LEDD were observed for the genes *GBA, LRRK2, PRKN, PLA2G6, ATP13A2,* and *VPS35* (Figure 4C).

**Figure 5:**
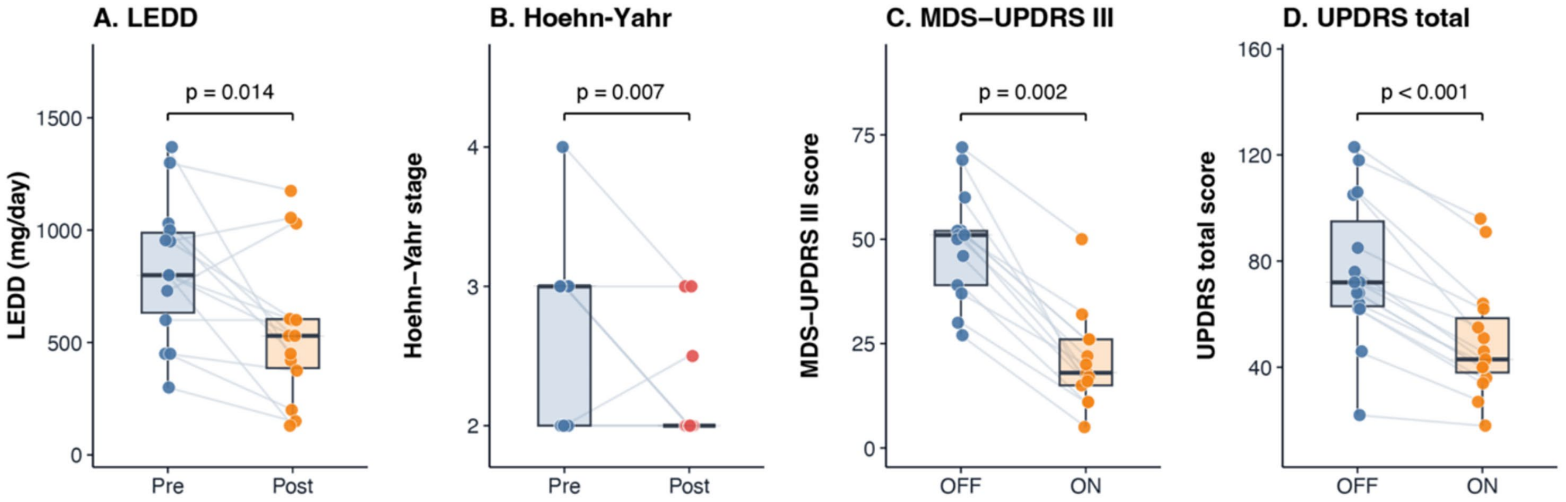
Contextual gene-listed EOPD DBS cohort. Paired plots summarize LEDD change **(A)**, H&Y ON-stage change **(B)**, preoperative MDS-UPDRS III ON/OFF response **(C)**, and preoperative UPDRS total ON/OFF response **(D)**.

Among 14 patients with paired medication data, the median LEDD decreased from 800.0 to 530.0 mg/day after DBS, corresponding to a median reduction of 35.2% (Wilcoxon *p* = 0.014, Figure 5A). H&Y stage in the ON-medication state improved from 3.0 to 2.0 (*p* = 0.007, Figure 5B). Preoperative medication-state testing showed robust levodopa responsiveness, with a median MDS-UPDRS III improvement of 63.3% when transitioning from OFF to ON medication (*p* = 0.002, Figure 5C). The UPDRS total ON/OFF response improvement was 39.6% (*p* < 0.001, Figure 5D).

## Discussion

This study describes two patients with SYNJ1-related EOPD who underwent DBS and were followed for 1.5 years. Both patients achieved significant reductions in postoperative medication. Case 1 experienced reduced medication requirements and improvements in dyskinesia and sleep after GPi-DBS, but did not show consistent improvement in MDS-UPDRS III scores at 1.5 years. Persistent motor fluctuations and neuropsychiatric symptoms indicate a partial and complex clinical response.

Case 2, who carried an intronic SYNJ1 variant, showed LEDD reduction and an improvement in the UPDRS-III score after STN-DBS. These contrasting outcomes support the clinical feasibility of DBS in selected cases of SYNJ1-related parkinsonism while cautioning against assuming uniform benefits across variant types.

The phenotype of Case 1 broadens the clinical spectrum of SYNJ1-related parkinsonism. The p.Thr657Ala substitution lies in the 5-phosphatase region of synaptojanin 1, distinct from previously described SAC1-domain variants, and was associated with levodopa-responsive EOPD, early motor complications, and rapid functional deterioration. The coexisting 46XY DSD is notable, but the absence of parkinsonism in the similarly affected sister suggests that no simple shared mechanism is involved; therefore, this association should be reported descriptively rather than causally. Case 2 highlights a different uncertainty: the c.1627 + 13 T > A intronic variant may affect splicing, but transcript-level confirmation is required before the molecular mechanism is considered established.

Target selection in these cases was clinically coherent. GPi-DBS was selected for Case 1 because dyskinesia was a dominant disability, whereas STN-DBS was selected for Case 2 in the setting of medication burden and parkinsonian motor symptoms. This distinction is important because genotype alone should not determine DBS candidacy or target. Current DBS practice emphasizes levodopa responsiveness, the disabling symptom profile, cognitive and psychiatric status, axial features, surgical risk, and patient goals (12–14). In genetic PD, these principles remain central, but variant-specific risks may modify expectations, particularly when cognitive, psychiatric, autonomic, or axial features are prominent.

Across 15 EOPD DBS patients with listed gene findings, DBS was associated with a median LEDD reduction of 35.2% and improvement in ON-medication H&Y stage. The gene spectrum was heterogeneous, including *GBA, LRRK2, PRKN, PLA2G6, ATP13A2, VPS35,* and several other candidate genes. These data are consistent with prior literature showing favorable DBS outcomes in selected PRKN, PINK1, and LRRK2-associated PD, while outcomes appear more variable in other genetic backgrounds. Additionally, there are concerns about cognitive vulnerability in some GBA-associated patients (15–17). The contextual cohort provides only descriptive clinical context. Its small size, heterogeneous gene findings, absence of a uniform variant-level classification, and non-comparative retrospective design preclude conclusions regarding gene-specific DBS efficacy. The cohort findings should therefore be interpreted as exploratory and hypothesis-generating.

While previous reports of SYNJ1-associated parkinsonism (PARK20) have primarily focused on mutations within the N-terminal SAC1 domain (e.g., the p. Arg258Gln variant), this case identifies a novel homozygous missense mutation (p. Thr657Ala) located specifically within the 5′-phosphatase domain. This is a critical distinction, as both domains are crucial to *SYNJ1* function. Unlike the well-documented p. Arg258Gln mutation located in the N-terminal Sac1 domain (Parkinson’s disease gene, Synaptojanin1, dysregulates the surface maintenance of the dopamine transporter), this variant resides within the 5′-phosphatase domain. This domain is responsible for dephosphorylating P1 (4, 5) P2, a critical step in shedding the clathrin coat from endocytic vesicles. *In silico* analysis suggests that this mutation is pathogenic and impairs domain function. Correspondingly, our patient presented with parkinsonism closely resembling idiopathic early-onset PD, but with the notable exceptions of rapid disease progression. This patient met all criteria for DBS implantation 4 years after the onset of symptoms (18). Despite a positive initial response to levodopa, her condition was complicated by motor fluctuations and dyskinesias, resulting in severe functional impairment. Importantly, she did not exhibit marked cognitive decline or autonomic dysfunction.

STN-DBS has demonstrated efficacy in patients with monogenic parkinsonisms, including *LRRK2*, *Parkin*, *PINK1*, *GBA* (10, 17, 19–21) and *SNCA* (22), yielding over 50% motor improvement (23–25), 60% reduction in levodopa-related motor complications (24, 25), 40 to 60% enhancement in quality of life (26), and a 50% reduction in the LEDD (27). However, clinical outcomes following STN-DBS can vary, with approximately half of patients developing stimulation-resistant symptoms such as gait impairment, postural instability, falls, cognitive impairment, and other non-motor deficits within 5 years of post-procedure (28). Notably, GBA mutation status is associated with cognitive decline, independent of age at PD onset and baseline Mattis Dementia Rating Scale (MDRS) scores (29). In contrast, patients with mutations in *LRRK2*, *PRKN*, *PINK1,* and *DJ-1* do not exhibit this association (20, 30).

## Conclusion

Our study reveals the phenotypic heterogeneity of *SYNJ1*-related EOPD caused by different variant types, including the first case of PARK20 presenting with 46, XY DSD. Exonic missense variants in functional domains correlate with rapid disease progression and severe motor complications, while deep intronic variants present a milder clinical course and a favorable surgical prognosis. Genetic sequencing for PD-related mutations is valuable in atypical PD, particularly in early-onset cases. DBS shows the potential as a therapeutic option for genetic atypical PD.

## Methods

### Case ascertainment and consent

The two cases were identified from patients with early-onset parkinsonism who were evaluated and treated at the First Affiliated Hospital of Shenzhen University, Shenzhen Second People’s Hospital. The diagnosis of Parkinson’s disease was established by movement disorder specialists in accordance with the Movement Disorder Society clinical diagnostic criteria (31). Written informed consent for publication of clinical details and images was obtained from the patients. The study and the contextual cohort analysis were approved under protocol [2022-005Q-01YJ].

### Genetic and clinical assessment

Clinical assessment included medical history, neurological examination, levodopa response testing, MDS-UPDRS or UPDRS scoring, Hoehn–Yahr staging, non-motor scales, cognitive screening, and neuroimaging, as available (32). Case 1 underwent Sanger sequencing of SYNJ1 and routine peripheral blood G-band karyotyping at 400-band resolution. Case 2 underwent genetic testing that identified SYNJ1 c.1627 + 13 T > A. Variant nomenclature should be verified against the final laboratory reports and reference transcript before submission.

### DBS surgery programming and follow-up

Case 1 underwent bilateral GPi-DBS with PINS L302 electrodes using robotic stereotactic assistance under general anesthesia, followed by implantation of a PINS G102RZ pulse generator in the left subclavicular region. DBS programming began 1 month after surgery using monopolar review in the medication-off state. The pulse width was initially set to 90 microseconds and the frequency to 150 Hz, with the voltage increased stepwise from 0 V to determine the therapeutic window. Case 2 underwent bilateral STN-DBS. Follow-up outcomes were assessed 1.5 years after DBS.

### Contextual EOPD DBS cohort and statistics

The contextual cohort was derived from a single-center analytic dataset of EOPD patients with motor onset at or before 50 years who underwent DBS and had listed gene findings. Gene entries were harmonized at the gene-symbol level. Core PD/parkinsonism genes were defined *a priori* as GBA, LRRK2, PRKN, PLA2G6, ATP13A2, and VPS35. Continuous variables were summarized using the mean and standard deviation and the median and interquartile range. Paired medication, staging, and ON/OFF medication comparisons used Wilcoxon signed-rank tests; exploratory group comparisons used Wilcoxon rank-sum tests. LEDD calculations should be verified against the local conversion protocol before submission (33).

## Data Availability

De-identified data supporting this report may be made available from the corresponding author upon reasonable request and after approval by the relevant institutional ethics committee. Public deposition is restricted because the data derives from identifiable rare disease cases and a small single-center clinical cohort.
