# Supplementary material for: Case Report: Deep brain stimulation in SYNJ1-related early-onset parkinsonism
Source: Front Med (Lausanne). 2026 Jul 13;13:1895449. doi: 10.3389/fmed.2026.1895449 (PMC13402543; doi:10.3389/fmed.2026.1895449)
Supplement: Supplementary file 1 [file Table_1.docx]

Supplemental Table S1. Listed gene spectrum in the contextual EOPD DBS cohort.

| Gene | Patients with gene | Category |
| --- | --- | --- |
| GBA | 2 (13.3%) | Core PD/parkinsonism gene |
| LRRK2 | 2 (13.3%) | Core PD/parkinsonism gene |
| PLA2G6 | 2 (13.3%) | Core PD/parkinsonism gene |
| ATP13A2 | 1 (6.7%) | Core PD/parkinsonism gene |
| PRKN | 1 (6.7%) | Core PD/parkinsonism gene |
| VPS35 | 1 (6.7%) | Core PD/parkinsonism gene |
| ABCA7 | 1 (6.7%) | Other/candidate gene |
| AMPD1 | 1 (6.7%) | Other/candidate gene |
| DYNC1H1 | 1 (6.7%) | Other/candidate gene |
| GIGYF2 | 1 (6.7%) | Other/candidate gene |
| NOS3 | 1 (6.7%) | Other/candidate gene |
| PNKD | 1 (6.7%) | Other/candidate gene |
| SETX | 1 (6.7%) | Other/candidate gene |
| TRPM2 | 1 (6.7%) | Other/candidate gene |
| TRPM7 | 1 (6.7%) | Other/candidate gene |
